# A Rare Location for Ossifying Fibroma: Temporal Bone Involvement

**DOI:** 10.7759/cureus.20637

**Published:** 2021-12-23

**Authors:** Yusuf Ç Kumbul, Mustafa Tüz, Vural Akın, Ayşenur Özen, Mehmet Kıran

**Affiliations:** 1 Department of Otorhinolaryngology and Head and Neck Surgery, Faculty of Medicine, Suleyman Demirel University, Isparta, TUR; 2 Department of Pathology, Faculty of Medicine, Suleyman Demirel University, Isparta, TUR

**Keywords:** excision, benign, head, ossifying fibroma, temporal bone

## Abstract

Benign fibro-osseous lesions of the craniofacial region are a diverse group of entities with overlapping histologic characteristics. One of these fibro-osseous lesions is ossifying fibroma and it is seen rarely in the head and neck region. Only a few cases of temporal bone involvement were reported in the literature. Patients with ossifying fibroma located in the temporal bone may have the following complaints: conductive hearing loss, swelling, localized pain, headache, and ear discharge. The lesion should be removed surgically and obtaining negative surgical margins is crucial to prevent any recurrence. A 29-year-old female patient who applied to our clinic with the complaint of a mass behind the left ear was treated, and the pathological diagnosis was an ossifying fibroma. In this study, a case of ossifying fibroma is presented.

## Introduction

Ossifying fibroma (OF) was first described by Mezel in 1872 [1]. OFs are benign fibro-osseous lesions that can develop at different sites in the body. They are rarely seen in the head and neck region, especially with temporal bone involvement. Instead, the mandible is the most common site of development [1-3]. The following complaints may be present with temporal bone involvement of OF: conductive hearing loss, swelling, localized pain, headache, and ear discharge [4]. OFs are seen as well-circumscribed masses with disorganized calcifications and disrupt the bone structures on radiological examinations [4]. For treatment, the lesion should be removed with negative surgical margins to prevent recurrence [2]. In this study, a case of OF located in the temporal bone was discussed in light of the literature.

## Case presentation

**Figure 1:**
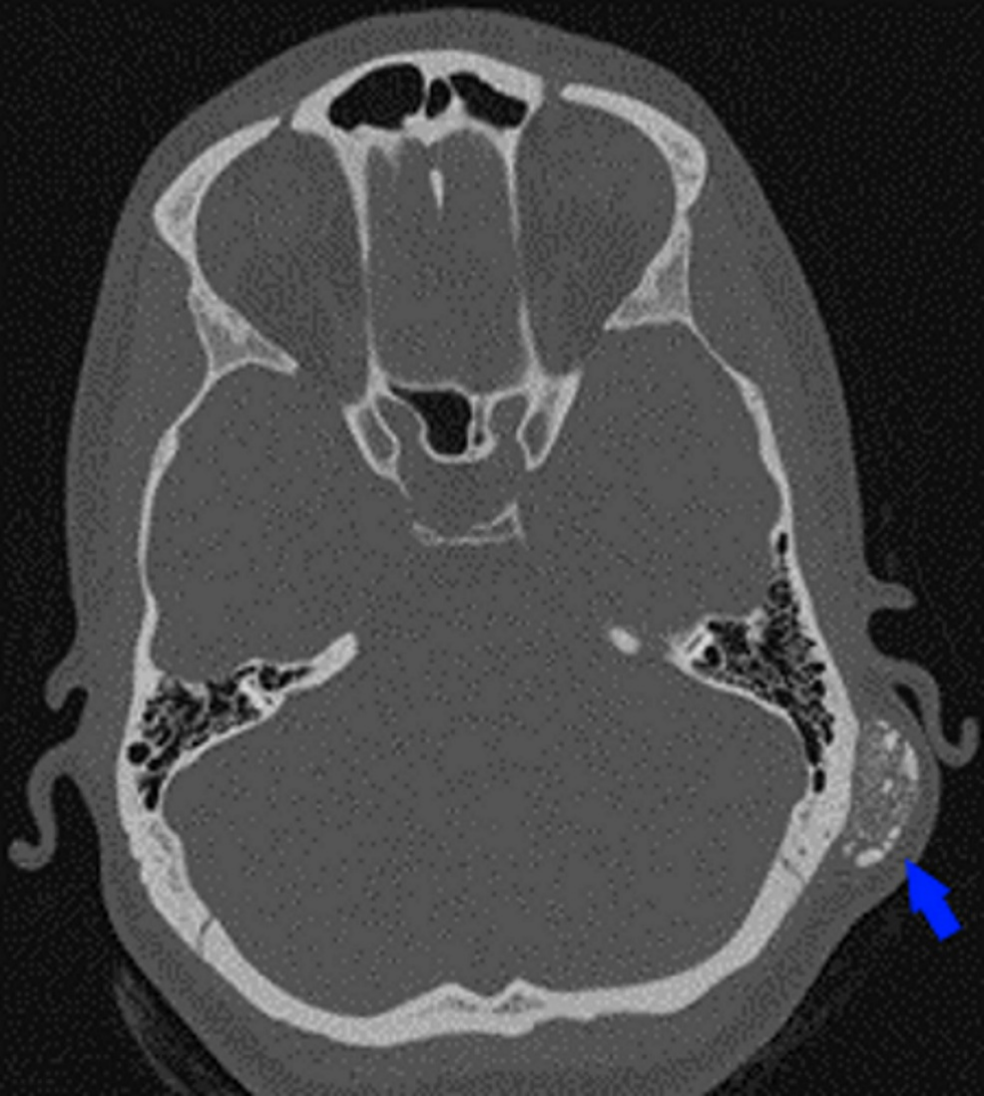
Temporal bone CT image of the lesion (blue arrow), axial section. CT: computed tomography.

A 29-year-old female patient was admitted to our clinic with a mass posterior to the left ear that had been enlarging slowly for about three years. The patient stated that her main complaint was cosmetic deformity. The patient had no history of head trauma or surgery. On physical examination, a rigid mass, approximately 30 × 25 mm in size, was palpated on the left mastoid process, with regular borders, fixed, without hyperemia and fever. Other than that, the patient's physical examination, audiological tests, complete blood count, and routine biochemical parameters were normal. The patient was evaluated with temporal bone computed tomography (CT). CT showed a well-circumscribed nodular lesion posterior of the left auricle, within subcutaneous fatty tissue, with a size of approximately 23 × 12 mm in axial sections, which did not destroy the adjacent bone structures, and contained extensive millimetric-sized amorphous calcifications (Figure 1).

**Figure 2:**
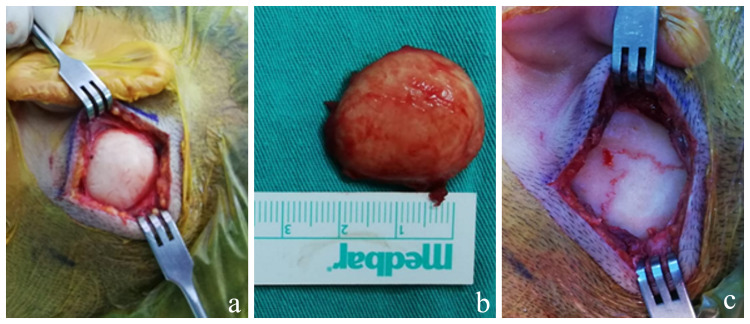
Intraoperative images of the lesion. (a) Intraoperative image after the incision, (b) post-excision image of the lesion, and (c) image of suture lines after excision.

Excision of the lesion under general anesthesia was planned. After the incision, it was noticed that the white-colored, well-circumscribed mass was attached to the temporal bone with a broad-based pedicle. The lesion and a part of the pedicle (due to the nature of the lesion) were excised. The remaining part of the pedicle was removed with the help of a drill, and the suture lines were visible (Figure 2).

**Figure 3:**
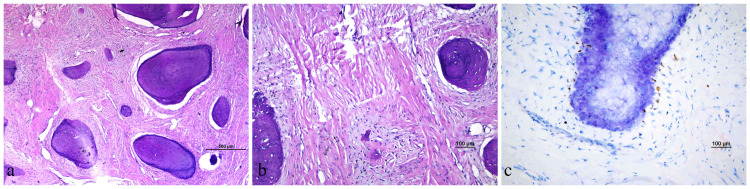
The histopathological and immunohistochemical examination of the lesion. (a) Hypocellular collagenized stroma and disorganized bone trabeculae that do not tend to integrate (Hematoxylin & Eosin, 40×). (b) Hypocellular collagenized stroma and a small area of bony trabeculae (Hematoxylin & Eosin, 100×). (c) No significant osteoblastic activity was observed in the SATB2 stained area (SATB2, 200×). SATB2: special AT-rich sequence-binding protein 2.

The patient was discharged on the first postoperative day, and there were no complications. Histopathological examination of the lesion reported OF. Microscopic examination revealed thick collagen bundles in the mass and disorganized bone trabeculae that did not tend to merge in the hypocellular stroma. No osteoclasts were seen in the bone tissue, and there was no significant osteoblastic activity (Figure 3). No recurrence was observed in the six-month follow-up of the patient. Informed consent form was obtained from the patient in order to use patient information in this case report.

## Discussion

OF was first described by Mezel in 1872 and is a rare fibro-osseous lesion of the head and neck region with a benign character and locally aggressive course [1-3,5]. Although OFs are mostly seen in the third and fourth decades, they can occur at any age and their frequency is similar between the sexes [1-3]. Female predominance is remarkable among young adults [2]. The case we present is compatible with the literature in terms of age and gender.

OFs in the head and neck region are most commonly seen in the mandible, followed by the maxilla [1,2]. It may rarely involve the orbit, paranasal sinuses, occipital bone, and temporal bone [1,3]. OFs, which can remain asymptomatic for many years, may cause various symptoms depending on the bone involved. OFs located in the temporal bone can cause conductive hearing loss, swelling, pain, headache, and ear discharge [4]. However, it should be kept in mind that OFs located extracranially on the temporal bone may also cause cosmetic deformity. Therefore, temporal bone OFs with the extracranial location may not cause the above-mentioned complaints.

For the clinical differential diagnosis of OFs located in the temporal bone, fibrous dysplasia, osteoma, exostosis, osteoblastoma, giant cell tumor, myelomas, eosinophilic granuloma, and metastatic lesions should be considered [6]. Among them, fibrous dysplasia is the most important because it is difficult to differentiate histopathologically [5]. Histopathologically, fibrous dysplasia consists of fibrous connective tissue containing bone trabeculae at different maturity stages. There is no osteoblastic activity in fibrous dysplasia. OFs are composed of acellular mineralized material and fibroblastic stroma containing lamellar bone [7]. Of the two slow-growing lesions, OFs are clinically more aggressive. In radiological examinations of OFs, well-defined borders and radiolucent images can be observed and there are disorganized calcifications within the tumor. However, a radiopaque lesion with irregular borders is seen in fibrous dysplasia [4]. As a result, the patient's history, physical examination, and radiological examinations will provide an idea about these lesions before the pathological examination.

In addition to conservative approaches such as enucleation and curettage, radical approaches can also be chosen for treatment. In a retrospective study in which eight case series were analyzed, the recurrence rate after surgical treatment was found to be 15.3%. It was stated that the recurrence rate in radical approaches is lower than in conservative approaches. In addition, it was revealed that 75% of recurrences are seen within the first year after surgery [2]. In our opinion, the rule that should be applied to prevent recurrence is to clearly reveal the borders of OF and to remove the bone with a drill until the healthy bone can be seen within these borders.

## Conclusions

OF is a very rare mass in the head and neck region. Temporal bone involvement especially is an exceptional case of OF. It should not be forgotten in the differential diagnosis of temporal bone masses and treatment should be surgical. The most confusing disease histopathologically in the differential diagnosis is fibrous dysplasia. OFs should be excised with broad surgical margins to prevent a recurrence.
